# A Brightness-Referenced Star Identification Algorithm for APS Star Trackers

**DOI:** 10.3390/s141018498

**Published:** 2014-10-08

**Authors:** Peng Zhang, Qile Zhao, Jingnan Liu, Ning Liu

**Affiliations:** 1 GNSS Research Center, Wuhan University, No.129 Luoyu Road, Wuhan 430079, China; E-Mails: fenix@whu.edu.cn (P.Z.); jnliu@whu.edu.cn (J.L.); 2 China Academy of Space Technology, Beijing 100094, China; E-Mail: lnrm0419@126.com

**Keywords:** star tracker, star ID, star magnitude, ZY-3, star brightness, *k*-*vector* search theory

## Abstract

Star trackers are currently the most accurate spacecraft attitude sensors. As a result, they are widely used in remote sensing satellites. Since traditional charge-coupled device (CCD)-based star trackers have a limited sensitivity range and dynamic range, the matching process for a star tracker is typically not very sensitive to star brightness. For active pixel sensor (APS) star trackers, the intensity of an imaged star is valuable information that can be used in star identification process. In this paper an improved brightness referenced star identification algorithm is presented. This algorithm utilizes the *k*-*vector* search theory and adds imaged stars' intensities to narrow the search scope and therefore increase the efficiency of the matching process. Based on different imaging conditions (slew, bright bodies, *etc.*) the developed matching algorithm operates in one of two identification modes: a three-star mode, and a four-star mode. If the reference bright stars (the stars brighter than three magnitude) show up, the algorithm runs the three-star mode and efficiency is further improved. The proposed method was compared with other two distinctive methods the pyramid and geometric voting methods. All three methods were tested with simulation data and actual in orbit data from the APS star tracker of ZY-3. Using a catalog composed of 1500 stars, the results show that without false stars the efficiency of this new method is 4∼5 times that of the pyramid method and 35∼37 times that of the geometric method.

## 1. Introduction

Star trackers are the most accurate attitude sensors for spacecraft attitude determination [1] and are typically used on space missions that have requirements for precise attitude knowledge. After taking photos of the stars, star trackers locate and identify the stars in the image. Using this information, the spacecraft's inertial attitude can be determined [2]. Current generation star trackers transfer the entire star image from the camera to the microprocessor, which then processes the whole image. Following star detection, the image coordinates of the stars are converted to incoming star vectors and each star is identified autonomously using an internal star catalog [3].

This paper presents an improved star ID method based on an existing search technique. This method employs the intensity of imaged stars to increase the matching efficiency. The rest of this paper is organized as follows. Sections 1∼3 introduce background information on the star trackers used for data collection as well as existing centroiding and star ID algorithms. Section 4 describes the new intensity-based method. Section 5 compares the performance of the new method with existing methods.

### 1.1. ZY-3 Star Trackers

There are two types of star cameras used in modern star trackers: Charge-Coupled Device (CCD) sensors and Active Pixel Sensors (APS). The APS use complementary metal oxide semiconductors (CMOS). As opposed to traditional CCD technology, the APS have the advantages of a higher dynamic range and higher blooming threshold, but are noisier because the photosensitivity of an APS pixel is non-homogeneous [4].

The ZY-3, China's first civilian three-line-array stereo mapping satellite, was launched on 9 January 2012. In order to meet accuracy requirement of 1 arc second, the ZY-3 satellite utilizes three APS star trackers, and multiple rate gyros [5]. This paper uses several raw star images from ZY-3′s star trackers to test the developed matching algorithm. The data was chosen from a star tracker when the satellite was in smooth flight at 7.61 km/s. The key parameters of the APS star trackers mounted on ZY-3 are given in Table 1.

### 1.2. Star ID

Star trackers typically operate in one of two operating modes: The lost-in-space (LIS) mode or tracking mode [2]. When a star tracker activates for the first time in space or when the previous attitude estimation fails, there is no information about the spacecraft's attitude. In these instances, a star tracker runs in a LIS mode which requires a search of the entire onboard star catalog for star identification.

When imaged stars have been successfully identified in a recent measurement, there is certitude about the spacecraft's attitude. In this case, star trackers can run in tracking mode, which requires a search of a small subset of the catalog based on the previous attitude estimate.

LIS is more complicated and time-consuming than the tracking mode due to the larger search range. Low earth orbit satellites experience more LIS situations because of the bright bodies (sun, moon, *etc.*) and space debris. These effects can lead to one of two scenarios which make matching impossible. The first scenario has a bright body obscuring all stars in the image, preventing any from being detected. In the second scenario, space debris leads to substantially more false detections which makes matching more difficult. Compared to APS star trackers, the traditional CCD based star trackers have narrower dynamic range [6], sensitivity [7], and are more sensitive to noise [8]. As a result, the intensity measurement from CCD-based star tracker is typically not used in existing matching algorithms [8,9].

Traditional CCD-based trackers have insufficient dynamic range to produce useful images when a target is closely approaching some bright bodies (asteroid, comet nucleus, *etc.*) [6]. In theory, APS star trackers have a larger dynamic non-blooming range than CCD-based star trackers. As a result, star intensity information from an APS-based star tracker can be used to identify the stellar magnitude of an imaged star. Results from the ZY-3 APS-based star trackers show that measurements of star intensity still vary up to 31%. However, despite this variation, the intensity of an imaged star can narrow the scope of the matching algorithms search within the catalog, decreasing the time required for execution. An efficient and robust star identification algorithm is presented in this paper that utilizes star brightness to narrow search scopes.

## 2. Relevant Matching Algorithms

Spratling *et al.* conducted a survey on star identification algorithms, in which they identified that star identification techniques typically fall into one of four categories, separated by two decisions [10]:

(a) Whether to use star brightness information.
(b) Whether to select any stars for a given star pattern.
(c) Whether to use ordinal information from brightness.
(d) Whether to use ordinal information from distance.

Spratling *et al.* mentioned that Ketchum [11] and Hong [12] used star brightness information in the lost-in-space algorithms. Ketchum uses the brightness of the two brightest stars to get the list of possible stars, while Hong uses the star brightness to get the ordered triple [9]. In this paper, the brightness of both the brightest star and two or three dimmest stars in an image is used to narrow the search scope.

In 1997, Mortari *et al.* proposed a *Search Less Algorithm*. The kernel of this technique is the *k*-*vector* method, a new range searching approach and faster than other search techniques. In 2004, Mortari also used this search method in the *Pyramid Star Identification Technique* which was successfully tested in a series of space missions: Draper Laboratory's “Inertial Stellar Compass”, and MIT's satellites HETE and HETE-2. In this paper, a modified *k*-*vector* search theory is employed to take advantage of magnitude information. The goal of the algorithm is to make full use of intensity information to improve *k*-*vector* search efficiency on the premise of credibility.

Two star ID methods are compared to test the performance of the new method. These methods are the *Pyramid Star Identification technique*, proposed by Mortari as stated above, and the *geometric voting algorithm*, which proposed by Michael Kolomenkin in 2006. A brief introduction of these two methods is given here.

### 2.1. Pyramid Star Identification

The *k*-*vector* search method of *Pyramid Star Identification technique* has larger search scopes than the method used in the present study. The *pyramid* algorithm attempts to find a unique triangle as the identification base to narrow the search scopes. To achieve this aim, Mortari *et al.* proposed a “smart” technique to determine the indices of subsequent star triangles. When scanning all possible observed star triads using three inner loops, and the star associated with the most external loop is a false star, most of the times spent is useless. The “smart” scan technique is used to avoid this problem. The resulting triangle sequence of this technique attempts to maximize the changes in the three indices that identify the triads.

A triangle is obtained and *k*-*vector* is used to access indices and establish a hypothesis for the cataloged indices for each star. Then *pyramid* scans the remaining stars to find one that further confirms the basic star triangle (*i*, *j*, *k*). When a fourth star is found, this demonstrates that these four stars are identified with a very high confidence. The basic star triangle (*i*, *j*, *k*) will then be used to identify the remaining stars as good ones when the stars confirm the basic star triangle or identify the measured image as a false star. If a confirmed fourth star is not found, then it chooses another “smart” combination of star indices making up another star triangle [13]. Prioritizing the establishment of a level of confidence is stressed as the basic philosophy of the authors in [13]. They prefer to report a star identification failure, rather than produce a star identification with low confidence.

### 2.2. Geometric Voting Algorithm

Presented by Michael Kolomenkin *et al.*, the geometric algorithm identifies the stars by utilizing their angular distances, instead of the polygons [8]. Catalog pairs vote for image star pairs with similar distances. As the angular distance is a symmetric relationship, each member of the catalog pair votes for each member of the image pair. The identity of the image star that receives the most votes is considered correct. In the preprocessing stage, the angular distances between all catalog star pairs are calculated. The list of inter star angles less than the threshold FOV are sorted by the angular distance. A distance table *T* is built according to this order. Every row in this table contains the distance *d* and the identities of the two stars *ID1* and *ID2.* Two steps are used to finish this identification process.

First and foremost is the voting step which confirms the possible identities of the stars. Every inter-angle δ*_ij_* of an image star pair is assumed to lie in the segment [θ*_ij_* − 3ε, θ*_ij_* + 3ε]. For all rows k in table *T* within this segment, a vote is cast for the identity of the two corresponding stars in the image. Binary search is used to find the first row in the table and a linear scan of the distance table is used to extract the rest of the rows. Assuming there are three image stars *s_i_*, *s_j_*, and *s_k_* where the inter angle between image stars *s_i_* and *s_j_* matches the distance between catalog stars *E* and *F*, then both image stars will get votes from *E* and *F*, meaning *E* and *F* are possible identities for each. If the inter angle between the image star *s_i_* and *s_k_* is equal to the distance between the catalog stars *E* and *G*, the image star *s_i_* will received two votes for being identified as catalog star *E*, one vote for being identified as catalog star *F*, and one vote for being identified as *G. s_i_* receives one vote for *E* and one vote for *F*, while *s_k_* receives one vote for *F* and *G*.

The second step is a validation step which is also a voting procedure. When the first step finished, for every image star, the identity that received the maximal number of votes is considered to be the exact identity. Meanwhile, if false stars creep into the image stars, their invalid identities lead to an erroneous attitude result. Every image star pair's inter angle is checked to make sure it is close to the distance between the stars with identifications from the catalog. If the distances are close, the two stars are voted. A simple clustering algorithm is used to recognize the correctly identified stars. Stars with incorrect identities receive an extremely small number of votes, whereas correctly identified stars will support each other.

## 3. Star Centroiding

Star centroiding accuracy directly affects the star ID process and the attitude results. In order to increase the ID success rate and meet the high accuracy demand, the centroids are required to have sub-pixel precision. The impulse response of an optical system is the point spread function (PSF). Since stars are effectively at an infinite distance, their corresponding images are typically approximated as the PSF of the sensor optics.

In an ideal imaging system, the size of the PSF can be smaller than a single pixel. However, many star trackers do not utilize diffraction limited optics, and consequently have PSFs much larger than a single pixel. A commonly used approximation for the shape of an imaged star is a symmetric Gaussian function.

In the star tracker image a star is distributed over several pixels. By using the center of mass (COM) method, the star centroid location estimate is improved over using the position of brightest pixel by up to an order of magnitude [14]. In our test we used two centroid determination methods: COM method and Gaussian interpolation method. The details of both methods are described in the subsequent subsections.

### 3.1. COM Method

The COM method is a very common approach used in computer vision and pattern recognition. COM is also widely used in star centroiding. Liebe [2] utilized a COM algorithm for centroiding onboard the JPL APS based star tracker. Once a pixel above a given threshold is detected, a rectangle window, region of interest (ROI), is aligned with the detected pixel in the center. Then the centroid is calculated based on the intensity and coordinates of the pixels in the window. Reference [2] gives the following equations:

(1) $$x_{cm}=\sum_{x=\text{ROIstart}+1}^{\text{ROIend}-1}\sum_{y=\text{ROIstart}+1}^{\text{ROIend}-1}\frac{x.\text{image}(\text{x},\text{y})}{DN}$$

(2) $$y_{cm}=\sum_{x=\text{ROIstart}+1}^{\text{ROIend}-1}\sum_{y=\text{ROIstart}+1}^{\text{ROIend}-1}\frac{y.\text{image}(\text{x},\text{y})}{DN}$$

*DN* is the background-subtracted pixel intensity in the ROI:

(3) $$DN=\sum_{x=\text{ROIstart}+1}^{\text{ROIend}-1}\sum_{y=\text{ROIstart}+1}^{\text{ROIend}-1}\text{image}(\text{x},\text{y})$$

### 3.2. Gaussian Interpolation Method

In 2007 Quine *et al.* proposed a Gaussian interpolation method for star centroiding. We give a short description about this method, since the PSF can be described as Gaussian function. Assuming that the CMOS pixel wells have an even intensity sensitivity across their active surface area, with a linear response, the intensity reading of a particular pixel *k* in the CMOS can be expressed as the integral over the active pixel area *K*:

(4) $$\begin{array}{ll} I_{k} & =I_{0}\frac{1}{\sqrt{2\pi }\sigma_{x}}\int_{x_{1}}^{x_{2}}\exp\left\{-\frac{{\left(\xi_{1}-x\right)}^{2}}{2\sigma_{x}^{2}}\right\}d\xi_{1}\frac{1}{\sqrt{2\pi }\sigma_{y}}\int_{y_{1}}^{y_{2}}\exp\left\{-\frac{{\left(\xi_{2}-y\right)}^{2}}{2\sigma_{y}^{2}}\right\}d\xi_{2}\\ & =\frac{I_{0}}{\pi }g(x,\sigma_{x},x_{1},x_{2})g(\text{y},\sigma_{y},{\text{y}}_{1},{\text{y}}_{2}) \end{array}$$

*g* is defined as the difference between two error functions:

(5) $$g(x,\sigma_{x},x_{1},x_{2})=\frac{\sqrt{\pi }}{2}\left\{\text{erf}(\frac{1}{\sqrt{2}\sigma_{x}}(x_{2}-x))-\text{erf}(\frac{1}{\sqrt{2}\sigma_{x}}(x_{1}-x))\right\}$$

(*x*_1_, *x*_2_) are the pixel boundaries, *x* is the centroid location in *x* direction, and σ*_x_* is the standard deviation of the incident light.

The intensity reading from two neighboring pixels or rows are compared:

(6) $$I_{1}=Ag(x,\sigma ,a,b),I_{2}=Ag(x,\sigma ,c,d)$$

where *x* is the Gaussian centroid offset; *a*, *b*, *c* and *d* are the pixel boundaries in *x*; and *A* is a constant. Eliminating *A* and linearizing *g* to second order, recovers a quadratic equation. If *I_2_* is the largest pixel measurement, the lower root of this quadratic equation provides the centroid offset.

### 3.3. Comparison of Two Methods

**Figure 1.**
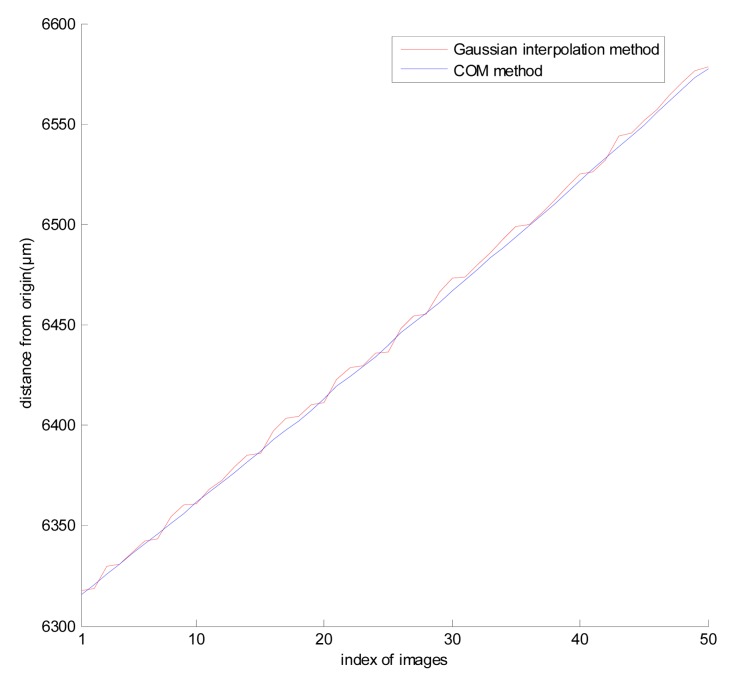
COM *vs.* Gaussian interpolation methods.

To compare the performance of both methods, they were tested on stellar images from a ZY-3 star tracker. Figure 1 shows the change of the distance between a 2.9 magnitude imaged star and the origin of star tracker coordinate in 50 successive images. The star tracker experienced a pure cross-axis slew (as opposed to an about-boresight roll). Therefore the centroid track of the imaged star should approximate a line. Linear regression is performed on both methods, and the coefficient of determination of the COM method is larger than that of the Gaussian interpolation method which indicates that the COM method is smoother than the Gaussian interpolation method. The possible reason is that COM method is a kind of weighted average method while the Gaussian interpolation method only uses two pixel information (one axis each), increasing the instability.

## 4. Brightness-Referenced ID Algorithm

Comparing with the previous two star ID methods, the main advantage of the proposed method is to adopt intensity information. Based on imaging conditions this algorithm runs in a three-star mode or a four-star mode which will be discussed in Section 4.3.

### 4.1. Basis of the Method

According to star magnitude's definition the illuminations of two adjacent magnitude stars *Lu_i_* and *Lu_i_*_+1_ have the following relationship:

> $$Lu_{i}=\sqrt[{5}]{100}\cdot Lu_{i+1}\approx 2.512\cdot Lu_{i+1}$$

Which means two stars, *n* and *m* magnitude respectively, have the following relationship [15]:

(8) $$\frac{Lu_{m}}{Lu_{n}}=2.512^{n-m}$$

The intensity of an imaged star (sum of the star area on image) is proportional to the illumination. An exponential function can be used to describe the relation between one star intensity *In_k_* to another, *In_m_*:

(9) $$In_{m}=In_{k}\cdot 2.512^{k-m}$$

where *k* and *In_k_* are the star magnitude and its corresponding intensity. *In_k_* is obtained by averaging the intensity values of an identified bright star in different images.

If an imaged star's intensity is larger than a certain value *M_t_* we can assume that the corresponding magnitude is brighter than three Mag. It is worth noting that the magnitude resolution for dimmer stars is limited by the performance of star trackers, since the intensity differences among dimmer stars are quite small.

More stars in the satellite mission catalog lead to increased searching time. Typically three stars are sufficient for attitude determination. However to get reliable star identification an additional star is needed [16].

**Figure 2.**
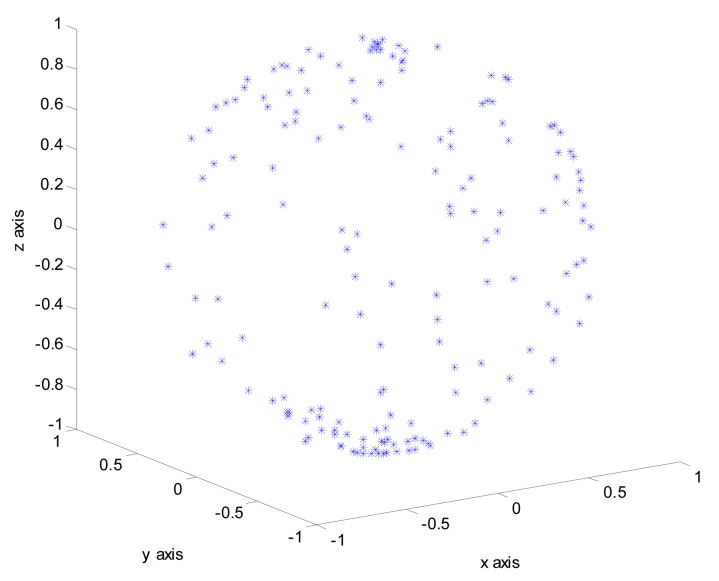
Normalized positions of Reference stars in the Celestial Sphere.

For the 20° × 20° wide FOV (field of view) star trackers, it is enough for star identification to detect down to the 4.5–5.0 magnitude [17]. As shown in Table 2, there are 1471 stars brighter than the 5.0 magnitude in HC. In most cases there are more than four stars in FOV during our research. 165 stars are brighter than three magnitude in the HC catalog, these bright stars can be considered as reference stars. The algorithm runs the three-star mode star ID once these stars appear in the FOV, which significantly improves efficiency compared with four-star mode. However as Figure 2 shows, these referenced stars are unevenly distributed across the celestial sphere, which means that the consistent appearance of these stars in the images is not guaranteed. There are three steps in the search process, each of them is introduced in the following subsections.

**Figure 3.**
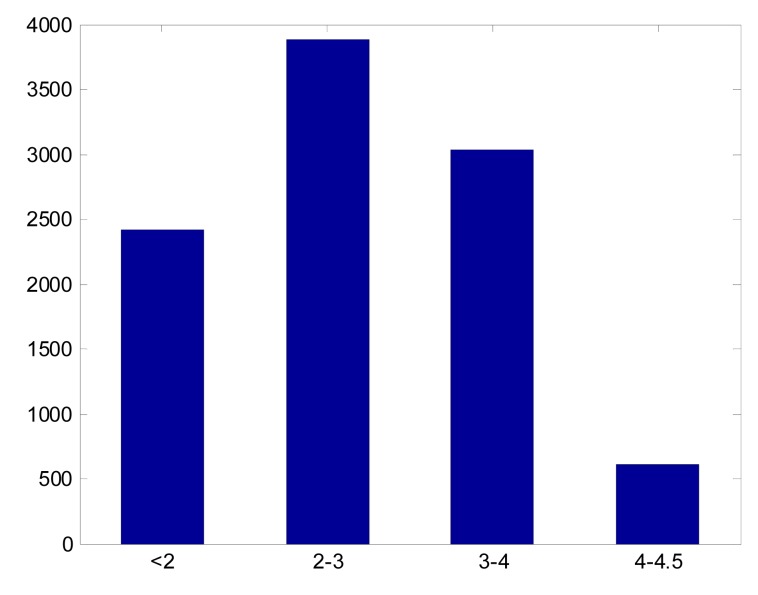
Histogram of the brightest star's magnitude distribution.

We analyzed 10,000 attitude orientations for a star tracker having a 20° × 20° FOV, the magnitude threshold set to 5.0 magnitude. These orientations are evenly distributed over the celestial sphere. In every case there were at least 4 stars in an image. Figure 3 shows the histogram of the brightest star's magnitude distribution in these 10,000 cases. The average magnitude difference between the brightest star and the third dimmest star in a single frame image is 2.16, and 97.52% of them were within the APS star trackers' magnitude detection accuracy. This analysis shows that in most orientations the ZY-3 star trackers will detect a star of magnitude three.

### 4.2. Modified k-Vector Search

The *k*-*vector* search method is a fast star database search technique, which Mortari presented in 1997 [18]. This method has been modified to include star intensity to improve matching performance.

#### 4.2.1. Star Pair Catalog

When the star catalogs (Bright Star Catalog, Tycho-2 catalog, Hipparcos catalog, SKY2000 Master Catalog and so on) [2,8,19] are used, the right ascension and declination of the star unit vectors in the Celestial Sphere Frame can be obtained. The Hipparcos Catalog, characterized by high precision and a near-real instrumental *Hp* photometry system, was adopted as the source for star unit vectors and visual magnitude.

If the star inter angle is smaller than FOV, the cosine of this angle is stored in ***P***. The elements of ***P*** are sorted in an ascending order:

(10) $$P=\left[\begin{array}{c} p_{1}\\ p_{2}\\ \vdots \\ p_{m} \end{array}\right],I=\left[\begin{array}{c} i_{1}\\ i_{2}\\ \vdots \\ i_{m} \end{array}\right],J=\left[\begin{array}{c} j_{1}\\ j_{2}\\ \vdots \\ j_{m} \end{array}\right],{\text{B}}_{I}=\left[\begin{array}{c} b_{i_{1}}\\ b_{i_{2}}\\ \vdots \\ b_{i_{m}} \end{array}\right],{\text{B}}_{J}=\left[\begin{array}{c} b_{j_{1}}\\ b_{j_{2}}\\ \vdots \\ b_{j_{m}} \end{array}\right]$$

Assuming there are m entries in ***P***, integer array ***I*** stores the brighter star identity *i_k_*, ***J*** stores the remain star identity *j_k_*, (where 1 ≤ k ≤ m), *i_k_* and *j_k_* corresponding to the star pair *p_k_*. *B_I_* and *B_J_* store the magnitude information which corresponds to ***I*** and ***J***. These five vectors construct the *star pair* catalog.

#### 4.2.2. Building the K Vector

**Figure 4.**
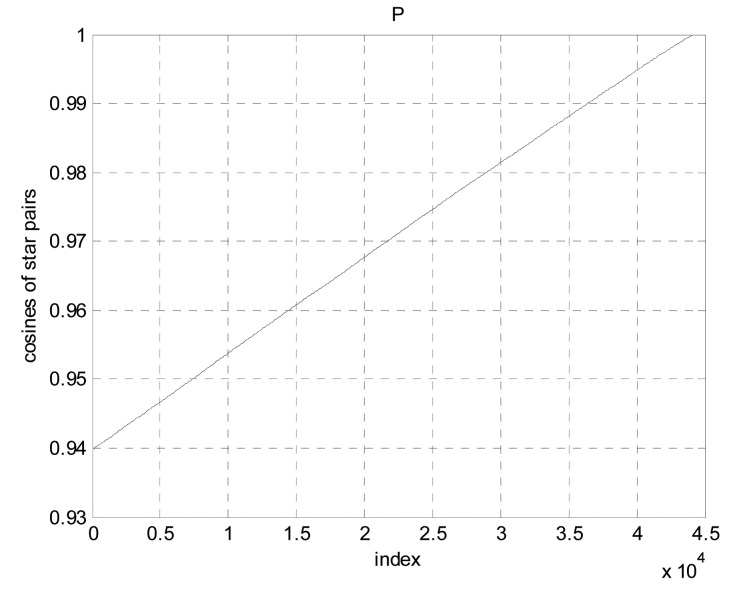
P as a function of indices.

Figure 4 shows the ***P*** as a function of indices 1,…, m. For the 1604 stars brighter than 5.0 magnitude in BSC, m is equal to 44,234.

Next the ***K*** vector must be constructed to contain the desired index being sought. Let a straight line connecting the two points (1, *p_1_*) and (*m*, *p_m_*). The following two equations are presented in [17], which describes how the straight line and ***K*** vector are built:

(11) $$y(\text{Ind})=a.\text{Ind}+b,a=\frac{p_{m}-p_{1}}{m-1},b=\frac{m\cdot p_{1}-p_{m}}{m-1}$$

As an element of ***K***, *i* must satisfy the following conditions:

(12) K(k)=i,P(i)≤y(k)=a⋅k+b<P(i+1)

where *k* = 2,…,*m* and the first element K(1) = 1. This algorithm presumes that *P*(i) is the nearest value less than or equal the value *y*(*k*). The indices of ***P*** are stored in ***K***.

#### 4.2.3. Evaluation of the Range

The search part of the *k*-*vector* search gets the range of the star inter angle from the *star pair catalog*. In the true star pair falling into the index range [*k_start_*, *k_end_*], the brighter star belongs to [*I_k_start__*,*I_k_end__*], the dimmer one belongs to [*J_k_start__*,*J_k_end__*], *k_start_* and *k_end_* are given by [15]:

(13) $$\{\begin{array}{c} k_{\text{start}}=K(j_{\text{bot}})+1\\ k_{\text{end}}=K(j_{\text{bot}}) \end{array}$$

where *j_bot_* and *j_top_* are also given by Mortari [17] as follows:

(14) $$\{\begin{array}{c} j_{\text{bot}}=\text{floor}\left(\frac{\cos(\theta +2\varepsilon )-b}{a}\right)\\ j_{\text{top}}=\text{ceil}\left(\frac{\cos(\theta -2\varepsilon )-b}{a}\right) \end{array}$$

θ is the star pair inter angle obtained from the star tracker, the precision of which is ε.The function *floor*(x) rounds the x to the nearest integer less than or equal to x, and *ceil*(x) rounds the x to the nearest integer greater than or equal to x.

### 4.3. Brightness-Referenced ID Process

After the *k*-*vector* search process, a small range of star pairs that need further confirmation are obtained. The two candidates in a contingent star pair extracted from the star image could be any star in the range [*I_k_start__*,*I_k_end__*] or [*J_k^start^_*,*J_k_end__*].

In the *Brightness*- *referenced ID algorithm* we do not use the image star intensity to determine the grade to which the star belongs, instead we only consider which is the brighter star in the candidate star pair. If the difference between two image stars' intensities is over the threshold γ, one star is considered brighter than the other, the brighter star falling into [*I_k^start^_*,*I_k_end__*] while the dimmer one belongs to [*J_k_start__*,*J_k_end__*].

When an image processing frame finishes, the *n* star-like points' coordinates *C_i_* and intensity information *Int_i_* is acquired (*i* = 1,…, *n*). The points *s_1_*,*s_2_*, …,*s_n_* are sorted in descending order as the magnitude decreases. In a case where *n* ≥ 4 and if the brightest star's intensity is beyond *M_t_* then the program runs in reference-star mode.

#### 4.3.1. Reference Star Mode

**Figure 5.**
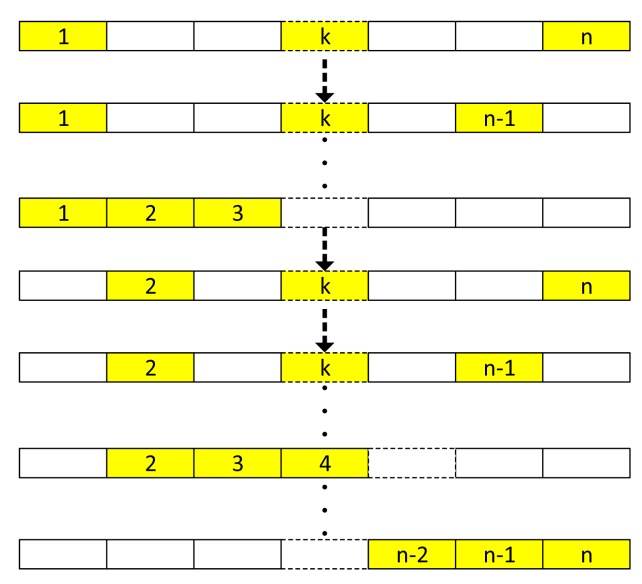
Logic diagram of a loop algorithm.

In this mode, the stars *s_i_*, *s_j_*, *s_k_* are sufficient for the identification. In an ideal situation, *s*_1_ and arbitrary two other stars can be chosen to finish this step. However, false stars exist in images that may be caused by dark current, hot pixel, or stars that are not in the catalog. When a star ID fails, another group of stars should be found. A sequential traversal of the *n* possible stars is not suitable in the *brightness*-*referenced ID algorithm*, because the intensity differences between the brightest star while the two other stars should be guaranteed within the detection accuracy threshold. A loop algorithm for this task is designed as follows:

Figure 5 shows this loop algorithm's diagram. This loop starts from the brightest star and two dimmest stars (*i* = 1, *j* = n, *k* = n − 1). The index *i* begins at 1 and counts to n − 2, *j* begins at n and counts to *i* + 2 and *k* begins at *j* − 1 and counts to *i* + 1. If *Int_i_* > *M_t_* then the loop continues; otherwise this ends the loop algorithm and it shifts to four-star mode. For the two pairs of possible stars *s_i_s_j_* and *s_j_s_k_*, using the *k*-*vector* search method, it acquires two index ranges [*k_s1_*, *k_e1_*] and [*k_s2_*, *k_e2_*]. Choosing stars that have magnitudes are over 3.0 and obtaining [*k′_s1_*, *k′_e1_*] and [*k′_s2_*, *k′_e2_*] further narrows the ranges, $R_{i}=\left[I_{k_{s1}^{\prime }},I_{k_{e1}^{\prime }}\right]\cap \left[I_{k_{s2}^{\prime }},I_{k_{e2}^{\prime }}\right]$. If *R_i_* is nonempty and unique it is assumed to be the correct star identity. The identity of *s_i_* then confirms the remaining two stars *s_j_* and *s_k_*. In case of a false star we still need examine *s_j_* d *s_k_*'s star catalog inter angle θ*_ij_* and determine if they match the measured angle δ*_ij_*. After the three stars are identified, use them to confirm the remaining image stars.

In the case n ≥ 4 if the brightest star's intensity is smaller than *M_t_* the program runs in the four-star mode.

#### 4.3.2. Four-Star Mode

When the reference stars do not appear, identification with three stars and two inter angles cannot sufficiently guarantee the result's uniqueness, while four stars and tree inter angles can significantly increase the possibility of uniqueness, in this situation the mismatching possibility is shown as follows [14]:

(15) $$\rho_{\text{mis}}=N{\left[(N-1)\sin(k\sigma )\right]}^{3}\sin\theta_{ij}\sin\theta_{ik}\sin\theta_{il}$$

where *N* is the amount of stars with magnitude less than *M*, *k*σ is the measurement precision, in most instances ρ*_mis_* ≪ 0.01.

The four-star mode loop algorithm has a similar principle as the three-star mode. The loop algorithm of the four-star mode nests the fourth loop into the previous three loop algorithm. This loop algorithm starts from four stars *s_1_*, *s_n_*, *s_n−1_*, *s_n−2_*. As previously discussed in this section, in most cases the intensity differential values of one image's brightest star *s_1_* and its three dimmest stars *s_n_*, *s_n−1_*, *s_n−2_* are within the detection accuracy. The three inter angles between *s_1_* and *s_n_*, *s_n−1_*, *s_n−2_* are *δ_1_, δ_2_, δ_3_* as calculated using Equations (13) and (14) we get the three ranges where the *p_1_* lies: [*I_k_s__*__1__,*I_k_e__*__1__], [*I_k_s__*__2__,*I_k_e__*___2___] and [*I_k_s__*__3__,*I_k_e__*__3__]. If the intersection set of the three ranges is nonempty it is assumed to be the correct star ID, else the loop enters into next circle. After the four stars' IDs are acquired, a checking step is still needed to avoid the false stars. In every cycle the brightest star and three dimmest stars of the remaining stars form the candidate group for the purpose of ensuring the intensity difference. The typical random frequencies for modern star trackers with four or more valid stars are smaller than 10^−7^, so matching four or more stars usually results in a nearly certain star identification, particularly if this event occurs during successive star identifications and the identified stars overlap [13].

**Figure 6.**
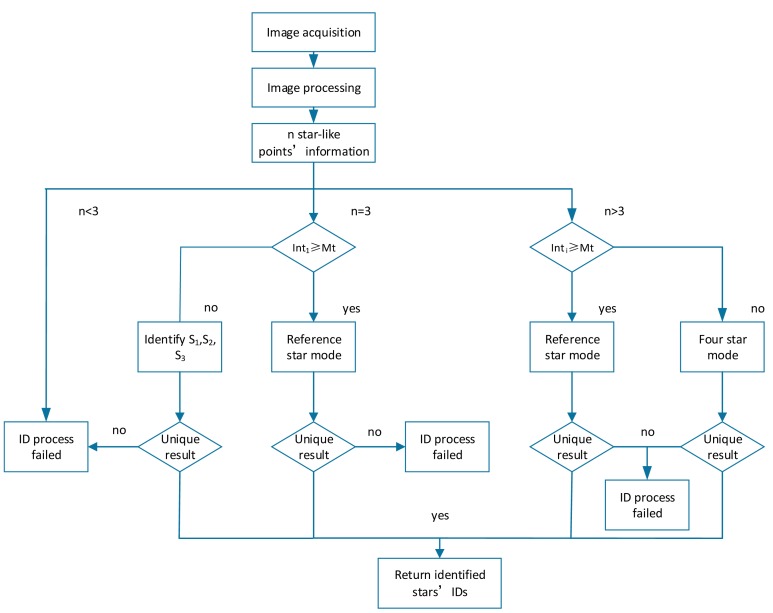
Flowchart of the brightness referenced ID algorithm.

If *n* = 3 (it is a rare situation for modern APS star trackers), the uniqueness of the triangle is the only item to be checked. However, even with the availability of a unique triangle, a comparison with previously identified stars should still be conducted. The entire process is shown as Figure 6:

## 5. Performance Comparison

For the purpose of comparing the performance of the three methods, 10,000 simulated orientations and 1000 ZY-3 APS star tracker images were selected. In addition to in orbit data, simulated data was used to compare the performance of the developed matching algorithm against that of the two existing methods. The threshold of catalog star magnitude was set to 5.0. All the algorithms were tested in MATLAB using i7-Q820, 1.73 GHz PC.

### 5.1. Simulations

**Figure 7.**
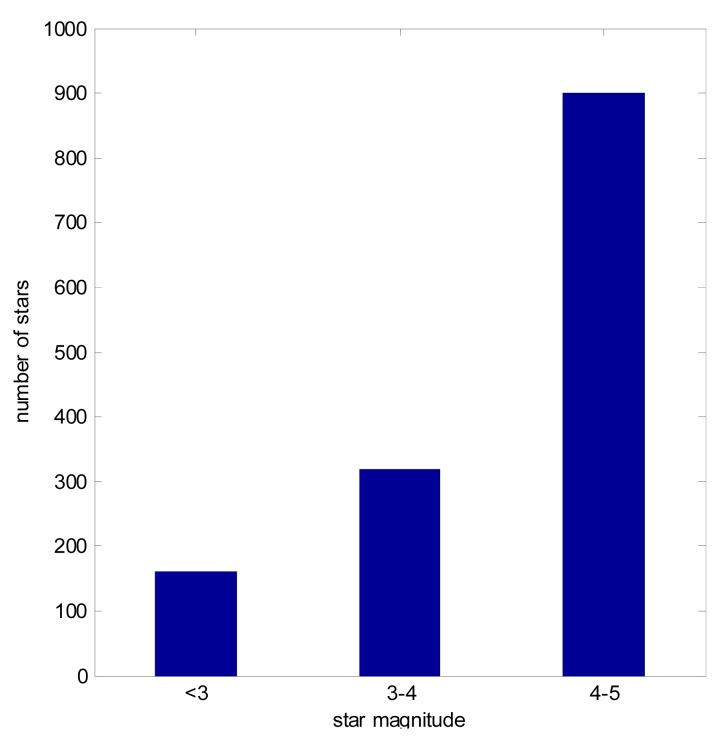
Brightness of simulated stars.

In simulations, a star tracker was simulated with 20° × 20° FOV, five arc seconds (3σ) accuracy and five arc seconds centroid accuracy. The image star intensity was simulated using Equation (9), assuming the magnitude uncertainty is 0.2 magnitude and *In_k_* is obtained from on orbit images. Every image contained at least three stars. The 10 brightest stars were chosen, at the most. The brightness of simulated stars are shown in Figure 7.

The simulations include three situations, *i.e.*, simulations without false star, simulations with at least four stars including one false star and simulations with at least five stars including one false star. The false star was simulated with random brightness and location in FOV. The results of simulations are shown in Table 3. The meanings of the parameters are given here. The time means execution time of the three algorithms which was measured using MATLAB function tic/toc. If the ID algorithms return the exact IDs this match can be called a success. If the returned IDs cannot match the exact IDs this match is called a bad match. If the algorithms cannot acquire star IDs it is called a failure.

For the first simulation situation, if at least four stars appeared in the image all of the three methods have delivered a correct result, whereas if three stars appeared, mismatching, particularly for the geometric voting method, may occur. In the present experiment, the *pyramid* method did not show its great advantage over the *binary*-*search* method that an independent of the star pairs' length *m*. This is because the amount of stars in the catalog was relatively small. For a fifteen hundred stars catalog the *binary*-*search* method needs a maximum of 16 lookups.

If a false star occurs randomly in the FOV, all three methods have to spend much more time finding the right solution, as shown in Table 3. Every image contained at least four stars (including a false star). There were 140 cases where there are four stars in an image among the 10,000 simulations. The 140 false stars were all dimmer than three mag, in this situation, the brightness-referenced method ran a four-star mode so ID failures or incorrect matches were inevitable. The geometric voting method faced a similar problem, a false star greatly disturbed the voting result when the count of stars was limited. Since the pyramid method attempted to find a candidate triangle first, there is still a chance to get the correct result. Consequently, the success rate of the brightness-referenced method dropped to 98.76% while the pyramid method had a high success rate.

Table 3 also shows that the success rate of all three methods increased to a higher level when there are at least five stars. The pyramid method is the most robust, it identified all of the simulations. In the 478 five stars cases our method failed 14 times and got 16 wrong results but it is still the most efficient method.

In the last two situations, the brightness-referenced method is not the most robust to false detections, and it has larger number of ID failures and bad matches than *pyramid* method. However, it is still the most efficient among these three methods.

### 5.2. In Orbit Data

We tested a random set of 1000 ZY-3 APS star tracker images which were selected from data from four different days. The results of the three methods for on orbit images are shown in Table 4. All in orbit images contain at least six stars brighter than five mag, the brightness-referenced method and pyramid method both identified all of the images. The geometric voting method failed when the count of false stars was almost the same as the actual stars. Coincidentally, in the first voting step false stars get the most votes, so if the satellite mission catalog choosing the bright stars, then the small data size will decrease this method's robustness.

## 6. Conclusions

CCD star trackers have a smaller dynamic range than CMOS star trackers. As a result, CMOS-based star trackers have the ability to more accurately measure star intensity, across a larger range. To make full use of this advantage, an improved star brightness-referenced star ID method was presented. Instead of using intensity to identify the coarse-grained magnitude of a star, bright stars can be used as reference stars in three-star mode and the intensity information is used to determine which one is brighter so as to narrow the search scope.

All simulations and tests with real data as represented in Tables 3 and 4 show that the proposed star ID method is at least twice as efficient than the pyramid method and eight times more than the geometric voting method. However, if false stars exist, the proposed method got more wrong results than the pyramid method. The in-orbit images show that some measured magnitudes of the stars are quite different from the corresponding catalog magnitude; the disagreement can be up to 0.7 magnitude (in most cases the measured magnitude is smaller than that in the catalog). In an image, the disagreement of 0.7 happened only on a dim star, making no difference in the identification of the brightest star. Therefore, the proposed method still works in this extreme case. However, this method will be invalidated if the number of stars is limited and the imaged star with the largest intensity is not the one with the brightest catalog magnitude.

The efficiency of the brightness-referenced method is significantly degraded when false stars appear brighter than a magnitude three star. If a direct relation between star magnitude and image intensity is established, the algorithm can be expected to be simpler and more robust. In the near future the advent of APS sensors with higher stability and accuracy will help make it possible.

## Figures and Tables

**Table 1.** Key Parameters of ZY-3′s APS Star Tracker.

**Field of View**	**20° × 20°**
Dimension	1024 × 1024 pixels
Pixel size	15 μm × 15 μm
Focal length	43.3 mm
Exposure time	125 ms∼500 ms
Accuracy (cross-boresight)	5 arcsec (3σ)
Stellar detection threshold	6.5 magnitude
Catalog size	≤5.5 magnitude
Update rate	4 Hz

**Table 2.** Number of HC Stars for Each Star Magnitude.

**Star Magnitude Range**	**Number of Stars**
<3	165
3–4	315
4–5	991
5–6	3088
6–7	9381

**Table 3.** Results of three methods with different situations.

**Scenarios**	**Parameters**	**Brightness-Referenced Method**	***Pyramid* Method**	**Geometric Voting Method**
Simulations with no false star	Success rate	99.82%	99.91%	98.72%
	Average time	0.77 ms	3.93 ms	28.87 ms
	ID failure	18	9	106
	Bad match	0	0	22

Simulations that included a false star with at least four stars	Success rate	98.76%	99.25%	97.87%
	Average time	5.12 ms	10.65 ms	43.38 ms
	ID failure	118	0	175
	Bad match	6	75	38

Simulations that included a false star with at least five stars	Success rate	99.70%	100%	99.14%
	Average time	4.78 ms	10.36 ms	44.27 ms
	ID failure	14	0	84
	Bad match	16	0	2

**Table 4.** Results of the tree methods with on-orbit data.

**Parameters**	**Brightness-Referenced Method**	***Pyramid* Method**	**Geometric Voting Method**
Success rate	100%	100%	99.2%
Average time	4.00 ms	13.49 ms	35.18 ms
ID failure	0	0	0
Bad match	0	0	8
